# Analyzing factors affecting positivity in drive-through COVID-19 testing: a cross-sectional study

**DOI:** 10.1186/s12985-024-02388-w

**Published:** 2024-05-14

**Authors:** Masahiko Mori, Kazuaki Yokoyama, Riri Sanuki, Fumio Inoue, Takafumi Maekawa, Tadayoshi Moriyama

**Affiliations:** 1 Department of Internal Medicine, Sasebo Memorial Hospital, Sasebo, Nagasaki, 858-0922 Japan; 2 Sasebo city medical association, Sasebo, Nagasaki, 857-0801 Japan; 3 Department of Health and Welfare, Sasebo city office, Sasebo, Nagasaki, 857-0042 Japan; 4 Sasebo city Health Center, Sasebo, Nagasaki, 857-0042 Japan; 5 Department of Surgery, Sasebo Memorial Hospital, Sasebo, Nagasaki, 858-0922 Japan; 6 Department of Surgery, Fukuoka Central Hospital, Fukuoka, Fukuoka 810-0022 Japan; 7 Department of Neurosurgery, Sasebo Memorial Hospital, Sasebo, Nagasaki, 858-0922 Japan

**Keywords:** COVID-19, Drive-through testing, Symptoms, Omicron, Sample collection technique, Contact history

## Abstract

**Background:**

Demand for COVID-19 testing prompted the implementation of drive-through testing systems. However, limited research has examined factors influencing testing positivity in this setting.

**Methods:**

From October 2020 to March 2023, a total of 1,341 patients, along with their clinical information, were referred from local clinics to the Sasebo City COVID-19 drive-through PCR center for testing. Association between clinical information or factors related to the drive-through center and testing results was analyzed by Fisher’s exact test and logistic regression models.

**Results:**

Individuals testing positive exhibited higher frequencies of upper respiratory symptoms; cough (OR 1.5 (95% CI 1.2–1.8), *p* < 0.001, q = 0.005), sore throat (OR 2.4 (95% CI 1.9-3.0), *p* < 0.001, q < 0.001), runny nose (OR 1.4 (95% CI 1.1–1.8), *p* = 0.002, q = 0.009), and systemic symptoms; fever (OR 1.5 (95% CI 1.1-2.0), *p* = 0.006, q = 0.02), headache (OR 1.9 (95% CI 1.4–2.5), *p* < 0.001, q < 0.001), and joint pain (OR 2.7 (95% CI 1.8–4.1), *p* < 0.001, q < 0.001). Conversely, gastrointestinal symptoms; diarrhea (OR 0.2 (95% CI 0.1–0.4), *p* < 0.001, q < 0.001) and nausea (OR 0.3 (95% CI 0.1–0.6), *p* < 0.001, q < 0.001) were less prevalent among positives. During omicron strain predominant period, higher testing positivity rate (OR 20 (95% CI 13–31), *p* < 0.001) and shorter period from symptom onset to testing (3.2 vs. 6.0 days, *p* < 0.001) were observed compared to pre-omicron period. Besides symptoms, contact history with infected persons at home (OR 4.5 (95% CI 3.1–6.5), *p* < 0.001, q < 0.001) and in office or school (OR 2.9 (95% CI 2.1–4.1), *p* < 0.001, q < 0.001), as well as the number of sample collection experiences by collectors (B 7.2 (95% CI 2.8–12), *p* = 0.002) were also associated with testing results.

**Conclusions:**

These findings underscore the importance of factors related to drive-through centers, especially contact history interviews and sample collection skills, for achieving higher rates of COVID-19 testing positivity. They also contribute to enhanced preparedness for next infectious disease pandemics.

**Supplementary Information:**

The online version contains supplementary material available at 10.1186/s12985-024-02388-w.

## Background

Since the first case of COVID-19 was reported on 15th January 2020, as of the end of April 2023, approximately 33.8 million people, which accounts for 27% of the population, have been confirmed to have a COVID-19 infection in Japan [1]. During this pandemic, there have been a total of eight waves of expanded infection periods, each characterized by a different predominant strain. The original (Wuhan) strain was predominant during the first to third waves, followed by the α (alpha) strain in the fourth wave, the δ (delta) strain in the fifth wave, and the ο (omicron) strain from the sixth to the eighth waves [1, 2]. To meet the demands for COVID-19 testing while reducing the burden on hospitals and ensuring the safety of healthcare workers, drive-through testing systems have been introduced at clinics, hospitals, and public places worldwide [3–7]. Taking advantage of its efficiency, extensive testing was performed at drive-through centers. However, a comprehensive evaluation, including factors associated with COVID-19 testing results, has not been well studied. Our objective in this study was to identify the factors associated with COVID-19 drive-through testing positivity in a cross-sectional study of 1,341 tests conducted in Japan.

## Methods

### Subjects and data Collection

This research received approval from the ethical review boards at Sasebo Memorial Hospital, Japan (approval number 2022-02). All procedures in this study were conducted in accordance with the ethical principles outlined in the Declaration of Helsinki. During the COVID-19 pandemic, in response to the need for PCR testing for local clinics, Sasebo city established a drive-through PCR center. Patients were initially seen at local clinics and then referred to the Sasebo city drive-through PCR center for testing along with their clinical information. Nasopharyngeal swab samples were collected by 49 attending medical doctors, and real-time PCR testing was conducted for diagnosis. A total of 1,341 patients were tested from October 2020 to March 2023. Background information on the patients, including sex, age, underlying health conditions, smoking history, estimated transmission route, and symptoms, was initially gathered by the local clinics where they were first seen and then summarized at the drive-through center. All symptoms and their frequencies are documented in Additional file 1.

### Statistical analysis

Statistical analysis was conducted using SPSS® 21.0 (IBM, Armonk, NY, USA). Differences in the frequency of background information and symptoms between COVID-19 positive and negative cases, as well as between subjects in the pre-omicron strain predominant period (from October 2020 to December 2021) and subjects in the omicron strain predominant period (from January 2022 to March 2023), were analyzed using Fisher’s exact test and false discovery rate analysis. Differences in age, symptom duration, and the number of symptoms between COVID-19 positive and negative cases, as well as between subjects during the pre-omicron strain predominant period and subjects during the omicron strain predominant period, were analyzed using Student’s t-test. Association between the number of sample collection among collectors and testing positivity was analyzed by linear regression model. A binary logistic regression model with multivariate analysis was applied to assess whether the identified factors independently influence the testing outcomes.

## Results

### Characteristics of subjects

Of the 1,341 enrolled subjects, 718 (54%) were female, and 623 (46%) were male (Table 1). The mean age at enrollment was 44 years (standard deviation [SD] 21, and range 0–99 years old). Two hundred four (15%) had a history of smoking, 373 (28%) had a diagnosed underlying health condition, and the mean number of symptoms before PCR testing was 3.1 (SD 1.3).

**Table 1:** Characteristics of subjects

			COVID-19			
		All	Positive	Negative	OR (95% CI)^a^	p
Number		1,341	477	864		
Child (≤ 17 years old)		159	49	110		
Adult (18–64 years old)		925	352	573		
Elderly adult (≥ 65 years old)		257	76	181		
Age^b^		44 ± 21	44 ± 19	44 ± 22		0.4
Sex	Female	718	261	457	1.1 (0.9–1.3)	0.5
	Male	623	216	407		
Smoking	Yes	204	63	141	0.8 (0.6–1.1)	0.1
	No	1,137	414	723		
Underlying health condition	Yes	373	98	275	0.6 (0.4–0.7)	< 0.001
	No	968	379	589		
Symptom duration (days)^b^		4.2 ± 3.6	3.0 ± 1.7	4.8 ± 4.4		< 0.001
Number of symptom^b^		3.1 ± 1.3	3.4 ± 1.4	2.9 ± 1.3		< 0.001

^a^OR (95% CI); Odds ratio (95% confidence interval range)

^b^; Mean ± standard deviation

Analyses between COVID-19 positives and negatives are also shown

### Differences between COVID-19 positives and negatives: Lower frequency of underlying health conditions, shorter period from symptom onset to testing, and higher number of symptoms among COVID-19 positives

First, we investigated whether there were significant differences in characteristic information between COVID-19 positives (*n* = 477 (36%)) and negatives (*n* = 864 (64%)) (Table 1). Among them, COVID-19 positives exhibited a lower frequency of underlying health conditions compared to negatives (odds ratio [OR] 0.6 (95% confidence interval range [CI] 0.4–0.7), *p* < 0.001). Furthermore, COVID-19 positives had a shorter period from symptom onset to testing (mean ± SD: 3.0 ± 1.7 days vs. 4.8 ± 4.4 days, *p* < 0.001), and a higher number of symptoms (mean ± SD: 3.4 ± 1.4 vs. 2.9 ± 1.3, *p* < 0.001) compared to COVID-19 negatives.

### COVID-19 infection and symptoms: higher frequency of upper respiratory and systemic symptoms, but lower frequency of gastrointestinal symptoms among COVID-19 positives

We next analyzed the differences in symptoms between COVID-19 positives and negatives (Table 2 and Additional file 1). A total of 32 symptoms were identified. Among these, COVID-19 positives experienced fever (OR 1.5 (95% CI 1.1-2.0), *p* = 0.006, q = 0.02), runny nose (OR 1.4 (95% CI 1.1–1.8), *p* = 0.002, q = 0.009), cough (OR 1.5 (95% CI 1.2–1.8), *p* < 0.001, q = 0.005), sore throat (OR 2.4 (95% CI 1.9-3.0), *p* < 0.001, q < 0.001), headache (OR 1.9 (95% CI 1.4–2.5), *p* < 0.001, q < 0.001), and joint pain (OR 2.7 (95% CI 1.8–4.1), *p* < 0.001, q < 0.001) significantly more frequently than negatives. Conversely, COVID-19 positives experienced diarrhea (OR 0.2 (95% CI 0.1–0.4), *p* < 0.001, q < 0.001) and nausea (OR 0.3 (95%CI 0.1–0.6), *p* < 0.001, q < 0.001) significantly less frequently than negatives. Between underlying health condition positives (*n* = 373) and negatives (*n* = 968), less frequency of sore throat (OR 0.6 (95% CI 0.4–0.7), *p* < 0.001, q < 0.001) and headache (OR 0.4 (95% CI 0.2–0.5), *p* < 0.001, q < 0.001) symptoms among underlying health condition positives compared to the negatives were identified (Additional file 1).

**Table 2:** Differences between COVID-19 positives and negatives in frequency of symptoms

		Frequency				
Symptom	COVID-19	+^a^	-^b^	OR (95% CI)^c^	p	q
Cough	Positive	227	250	1.5 (1.2–1.8)	< 0.001	0.005
	Negative	330	534			
Runny nose	Positive	238	239	1.4 (1.1–1.8)	0.002	0.009
	Negative	355	509			
Fever (≥ 37.0℃)	Positive	390	87	1.5 (1.1-2.0)	0.006	0.02
	Negative	648	216			
Headache	Positive	130	347	1.9 (1.4–2.5)	< 0.001	< 0.001
	Negative	144	720			
Joint pain	Positive	62	415	2.7 (1.8–4.1)	< 0.001	< 0.001
	Negative	45	819			
Sore throat	Positive	235	242	2.4 (1.9-3.0)	< 0.001	< 0.001
	Negative	250	614			
Nausea	Positive	9	468	0.3 (0.1–0.6)	< 0.001	< 0.001
	Negative	57	807			
Diarrhea	Positive	9	468	0.2 (0.1–0.4)	< 0.001	< 0.001
	Negative	69	795			

^a^+; indicates presence of a symptom

^b^-; indicates absence of a symptom

^c^OR (95% CI); Odds ratio (95% confidence interval range)

Significant differences (*p* < 0.05 by Fisher’s exact test and q < 0.1 by false discovery rate analysis) in frequency of symptoms are shown. Results for all symptoms are shown in Additional file 1

These data suggest differences between COVID-19 positives and negatives in the development of certain COVID-19-related symptoms, particularly a higher frequency of upper respiratory and systemic symptoms. When evaluating patients with gastrointestinal symptoms, consideration of symptoms derived from diseases other than COVID-19 infection may be warranted.

### Omicron vs. pre-omicron: higher testing positivity rate, shorter period from symptom onset to testing, and higher frequency of upper respiratory and systemic symptoms during omicron strain predominant period compared to pre-omicron strain predominant period

We next analyzed the differences between pre-omicron strain predominant period (from October 2020 to December 2021) and omicron strain predominant period (from January 2022 to March 2023) (Table 3). Omicron strain predominant period had a higher positive rate in testing (51% vs. 5.0%, OR 20 (95% CI 13–31), *p* < 0.001), younger age distribution (43 ± 20 years old vs. 46 ± 22 years old, *p* = 0.005), less frequency of subjects with underlying health condition (24% vs. 35%, OR 0.6 (95% CI 0.5–0.7), *p* < 0.001), shorter period from symptom onset to testing (2.9 ± 1.4 days vs. 5.3 ± 3.7 days, *p* < 0.001), and more number of symptom (3.1 ± 1.4 vs. 2.9 ± 1.3, *p* = 0.04). In terms of symptoms, subjects in the omicron strain predominant period experienced runny nose (OR 1.3 (95% CI 1.1–1.7), *p* = 0.02, q = 0.09), sore throat (OR 3.7 (95% CI 2.7–4.6), *p* < 0.001, q < 0.001), headache (OR 1.8 (95% CI 1.4–2.5), *p* < 0.001, q < 0.001), and joint pain (OR 1.8 (95% CI 1.1–2.8), *p* = 0.01, q = 0.05) significantly more frequently than subjects in the pre-omicron strain predominant period (Table 4 and Additional file 3). Conversely, subjects in the omicron strain predominant period experienced respiratory distress (OR 0.5 (95% CI 0.3–0.9), *p* = 0.01, q = 0.05), nausea (OR 0.5 (95% CI 0.3–0.8), *p* = 0.008, q = 0.05) and taste disorder (OR 0.1 (95% CI 0.06–0.3), *p* < 0.001, q < 0.001) significantly less frequently than positives in the pre-omicron strain predominant period.

**Table 3:** Differences between omicron strain predominant period and pre-omicron strain period

		Omicron	Pre-omicron	OR (95% CI)^a^	*p*
PCR testing positive rate		51% (454/882)	5.0% (23/459)	20 (13–31)	< 0.001
Age^b^		43 ± 20	46 ± 22		0.005
Sex	Female	483	235	1.2 (0.9–1.4)	0.2
	Male	399	224		
Smoking history	Yes	124	80	0.8 (0.6–1.1)	0.1
	No	758	379		
Underlying health condition	Yes	212	161	0.6 (0.5–0.7)	< 0.001
	No	670	298		
Time from symptom onset to testing (days)^b^		3.2 ± 2.4	6.0 ± 5.0		< 0.001
Number of symptom^b^		3.1 ± 1.4	2.9 ± 1.3		0.04

^a^OR (95% CI); Odds ratio (95% confidence interval range)

^b^; Mean ± standard deviation

**Table 4:** Differences between Omicron period and Pre-omicron period in frequency of symptoms

		Frequency				
Symptom	COVID-19	+^a^	-^b^	OR (95% CI)^c^	p	q
Runny nose	Omicron	411	471	1.3 (1.1–1.7)	0.02	0.09
	Pre-omicron	182	277			
Sore throat	Omicron	398	484	3.7 (2.7–4.6)	< 0.001	< 0.001
	Pre-omicron	87	372			
Headache	Omicron	208	674	1.8 (1.4–2.5)	< 0.001	< 0.001
	Pre-omicron	66	393			
Joint pain	Omicron	82	800	1.8 (1.1–2.8)	0.01	0.05
	Pre-omicron	25	434			
Respiratory distress	Omicron	41	841	0.5 (0.3–0.9)	0.01	0.05
	Pre-omicron	38	421			
Nausea	Omicron	33	849	0.5 (0.3–0.8)	0.008	0.05
	Pre-omicron	33	426			
Taste disorder	Omicron	10	872	0.1 (0.06–0.3)	< 0.001	< 0.001
	Pre-omicron	38	421			

^a^+; indicates presence of a symptom

^b^-; indicates absence of a symptom

^c^OR (95% CI); Odds ratio (95% confidence interval range)

Significant differences (*p* < 0.05 by Fisher’s exact test and q < 0.1 by false discovery rate analysis) in frequency of symptoms are shown. Results for all symptoms are shown in Additional file 3

### Drive-through center factors and COVID-19 testing: the importance of interviewing about estimated transmission route and nasopharyngeal swab sample collection technique in drive-through COVID-19 PCR testing

As additional factors associated with COVID-19 PCR testing, we analyzed the associations between the estimated transmission route or the experience of sample collection among collectors and the PCR testing results.

In the analysis between the estimated transmission route and PCR testing, COVID-19 positives reported the presence of an estimated transmission route (OR 2.5 (95% CI 2.0-3.2), *p* < 0.001, q < 0.001) significantly more often than negatives (Table 5). Specifically, contact with infected persons at home (OR 4.5 (95% CI 3.1–6.5), *p* < 0.001, q < 0.001) and office or school (OR 2.9 (95% CI 2.1–4.1), *p* < 0.001, q < 0.001) were identified as significantly associated with COVID-19 positivity. These results suggest the importance of doctors conducting interviews about estimated transmission routes prior to making decisions regarding PCR testing.

**Table 5:** Association between estimated transmission route and COVID-19 testing

	COVID-19				
	Positive	Negative	OR (95% CI)^a^	p	q
Estimated transmission route					
Yes	233	241	2.5 (2.0-3.1)	< 0.001	< 0.001
Unknown	244	623			
Home	93	53	4.5 (3.1–6.5)	< 0.001	< 0.001
Unknown	244	623			
Office or school	84	74	2.9 (2.1–4.1)	< 0.001	< 0.001
Unknown	244	623			
Travel history	34	70	1.2 (0.8–1.9)	0.4	0.4
Unknown	244	623			
Contact with persons from outside	11	29	0.9 (0.5-2.0)	1	0.8
Unknown	244	623			
Eating out	11	16	1.8 (0.8–3.8)	0.2	0.2
Unknown	244	623			

^a^OR (95% CI); Odds ratio (95% confidence interval range).

P values by Fisher’s exact test and q values by false discovery rates are shown

**Fig. 1:**
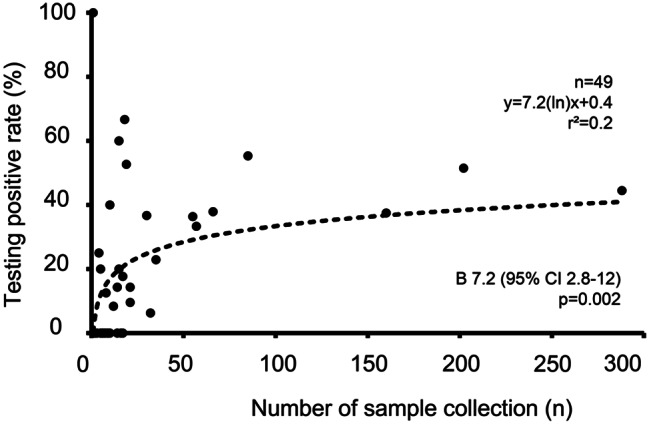
Association between sample collection experience and COVID-19 PCR testing result. Nasopharyngeal swab sample was collected by 49 collectors in charge, and association between the number of sample collection among collectors and PCR testing positive rate was analyzed

Next, we analyzed the associations between the number of sample collection among collectors and PCR testing results. An increase in the testing positivity rate was identified based on the collectors’ experience in sample collection among 49 collectors (B 7.2 (95% CI 2.8–12, *p* = 0.002)) (Fig. 1). This result emphasizes the importance of acquiring the technique for nasopharyngeal swab sample collection in a drive-through testing system.

### Multivariate analysis: various factors contributing to drive-through nasopharyngeal COVID-19 PCR testing

**Fig. 2:**
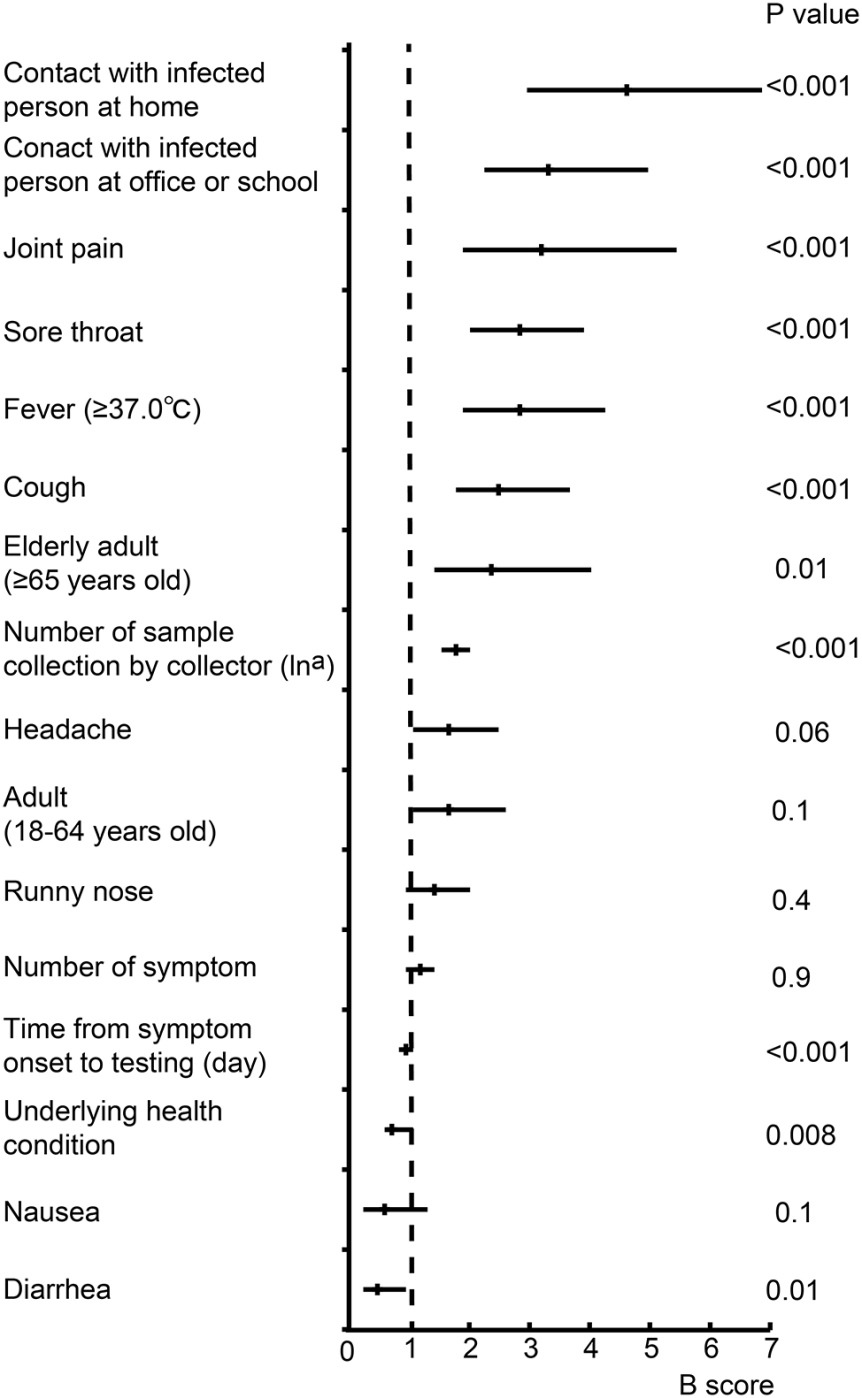
Factors associated with drive-through nasopharyngeal COVID-19 PCR testing positivity. Multivariate binary logistic regression model analyses, with B scores and their 95% confidence interval ranges are shown. Variables with significance (*p* < 0.05) in univariate analysis were applied to the multivariate analysis. Variables are shown from the highest B score to lower. Results for univariate analyses are shown in Additional file 4. ln^a^; log natural

Finally, to confirm whether the significant factors identified above (Tables 1, 2 and 5; Fig. 1) were independently associated with COVID-19 PCR testing, multivariate analysis was performed. The analysis revealed that estimated transmission route at home (B 3.9 (95% CI 2.5–5.8), *p* < 0.001) was the strongest variable independently associated with testing positivity. Estimated transmission route at office or school (B 2.8 (95% CI 1.9–4.2), *p* < 0.001), joint pain (B 2.7 (95% CI 1.6–4.6), *p* < 0.001), sore throat (B 2.4 (95% CI 1.7–3.3), *p* < 0.001), fever (B 2.4 (95% CI 1.6–3.6), *p* < 0.001), cough (B 2.1 (95% CI 1.5–3.1), *p* < 0.001), elderly adult (age ≥ 65 years old) patients (B 2.0 (95% CI 1.2–3.4), *p* = 0.01), number of sample collection by collectors (B 1.5 (95% CI 1.3–1.7), *p* < 0.001), time from symptom onset to testing (day) (B 0.8 (95% CI 0.7–0.9), *p* < 0.001), underlying health condition (B 0.6 (95% CI 0.5–0.9), *p* = 0.008), and diarrhea (B 0.4 (95% CI 0.2–0.8), *p* = 0.01), were identified as significant factors independently associated with PCR testing results (Fig. 2 and Additional file 4). These findings suggest that not only symptoms but also background information such as estimated transmission route, underlying health condition, age of patients, as well as the technique of sample collection, are important for the effectiveness of the nasopharyngeal swab drive-through COVID-19 PCR testing system.

## Discussion

This study systematically investigated the associations between host, pathogen, and drive-through center factors and PCR testing in a cross-sectional study of drive-through COVID-19 PCR testing. We observed a significantly higher frequency of upper respiratory and systemic symptoms, a lower frequency of gastrointestinal symptoms and underlying health conditions, and a shorter period from symptom onset to testing in COVID-19 positive individuals compared to negatives. Moreover, we found a higher COVID-19 positive rate in testing, a higher frequency of upper respiratory and systemic symptoms, a lower frequency of taste disorder, and a shorter period from symptom onset to testing during the omicron strain predominant period compared to the pre-omicron strain predominant period. Additionally, we identified the importance of conducting interviews about estimated transmission routes prior to testing and employing appropriate techniques for nasopharyngeal swab sample collection in drive-through testing.

Upper respiratory symptoms and systemic symptoms are known to be among the most frequent symptoms in COVID-19 infected patients [7–10], and our results showing a higher frequency of fever, runny nose, cough, sore throat, headache, and joint pain among COVID-19 positive individuals compared to negatives are consistent with these findings. The presence of underlying health conditions is known to be a factor associated with the worse progression and severity of COVID-19 disease [11–14], as well as an increased risk of adverse effects following COVID-19 vaccination, especially among individuals with a history of allergies [15–17]. However, we identified a lower frequency of underlying health conditions among COVID-19 positive individuals in this study, which was consistent with a report from the USA [18]. A reduced frequency of sore throat and headache symptoms was noted among individuals with underlying health conditions compared to those without. Given that sore throat and headache were one of the predominant COVID-19-associated symptoms in this study, the onset of symptoms attributable to underlying health conditions might result in negative PCR test results, consequently lowering the overall positivity rate among this subgroup. Although Japan does not have a system of local responsible medical doctors similar to general practitioners in the UK or Australia, médecin traitant in France, or hausarzt in Germany, patients with underlying health conditions first visit local clinics and are then referred to regional drive-through COVID-19 testing centers for diagnosis. Therefore, the decision of COVID-19 testing by local doctors, taking into account symptoms derived from underlying health conditions, becomes important for the further effectiveness of drive-through testing.

In PCR testing during the omicron strain predominant period, we observed an approximately 10-fold higher positive rate, a higher frequency of upper respiratory and systemic symptoms, and a shorter period from symptom onset to testing compared to the pre-omicron strain predominant period. This can be attributed to the higher viral replication capacity in the upper respiratory tract and a shorter window period associated with the omicron strain, as compared to other strains [19]. The increased viral replication capacity in the upper respiratory tract would contribute to the higher frequency of sore throat symptoms [20], earlier symptom recognition, and patient presentation to healthcare providers, leading to a higher positive rate in PCR testing under conditions of higher viral load [21, 22], as compared to infections with other strains. These findings underscore the importance of considering the virological and clinical characteristics differences among COVID-19 strains when evaluating and managing patients.

Besides symptoms, the information regarding estimated transmission routes was identified as a significant factor associated with PCR testing positivity. Among these routes, transmission at home emerged as the strongest variable, even stronger than symptoms in multivariate analysis. This highlights the importance of doctors or drive-through center conducting interviews to gather information about estimated transmission routes prior to making decisions regarding PCR testing.

Another host factor associated with PCR positivity independent of symptoms was the age of patients, with higher positivity among elderly adult patients. Immunosenescence, characterized by a reduced immune response against novel pathogen or vaccine, may contribute to the higher viral load and PCR positivity in testing among elderly adults, and this could be considered as its mechanism [23, 24]. Indeed, a positive correlation between age and viral load among COVID-19-infected adults was previously reported [25, 26].

The number of sample collection by collectors was identified as a unique drive-through factor associated with PCR positivity in this study. Although the samples collected differ, previous reports have emphasized the importance of sample collection by trained personnel, which showed higher sensitivity and specificity in COVID-19 testing for nasopharyngeal swab samples compared to self-collected oral or anterior nasal swab samples [27, 28]. Training on proper nasopharyngeal swab sample collection would be crucial for maintaining a stable diagnostic system and ensuring the safety of sample collectors in the drive-through system. Finally, regarding the shorter period from symptom onset to testing and the higher testing positivity, some patients had to wait until the next day for testing due to center’s schedules, such as seeing doctors late at night and having the test scheduled for the following day. Although partially, the center’s schedule also could be one of the factors associated with positivity.

As a limitation of this study, overlapping of confidence interval ranges among results was noted. This limitation arouse due to analyses conducted with a small sample size in this cross-sectional study. To ensure precision in future studies, results obtaining results without overlapping confidence interval ranges in larger sample sizes would be warranted. Secondly, the detection of viral strains in each PCR-positive case through genome sequencing was not performed. In order to conduct precise analyses on the associations between viral strains and symptoms, information about the viral strains would be necessary. Additionally, information regarding the COVID-19 vaccination history of the patients was not available. The vaccination history information may have had an impact, particularly on the associations between symptoms and PCR positivity, as there have been reports of fewer symptoms among vaccinated individuals who were infected with COVID-19 [29, 30].

## Conclusions

In conclusion, this study identified several factors associated with drive-through COVID-19 PCR testing positivity. Notably, specific drive-through center factors such as interviews regarding estimated transmission routes and swab sample collection technique by collectors were identified as unique factors in this study. The recognition of these distinctive factors associated with drive-through testing positivity presents an opportunity to gain a better understanding of the nature of COVID-19 infection, and prepare for the next pandemic of infectious diseases.

### Electronic supplementary material

Below is the link to the electronic supplementary material.

Supplementary Material 1

Supplementary Material 2

Supplementary Material 3

Supplementary Material 4

## Data Availability

The datasets used and/or analyzed during the current study are available from the corresponding author on reasonable request.
